# Differentiating Two Closely Related *Alexandrium* Species Using Comparative Quantitative Proteomics

**DOI:** 10.3390/toxins13010007

**Published:** 2020-12-23

**Authors:** Bryan John J. Subong, Arturo O. Lluisma, Rhodora V. Azanza, Lilibeth A. Salvador-Reyes

**Affiliations:** 1 Marine Science Institute, University of the Philippines- Diliman, Velasquez Street, Quezon City 1101, Philippines; aolluisma@up.edu.ph (A.O.L.); rvazanza@up.edu.ph (R.V.A.); 2 Department of Chemistry, The University of Tokyo, 7-3-1 Hongo, Bunkyo City, Tokyo 113-8654, Japan

**Keywords:** proteomics, *Alexandrium*, harmful algal bloom (HAB)

## Abstract

*Alexandrium minutum* and *Alexandrium tamutum* are two closely related harmful algal bloom (HAB)-causing species with different toxicity. Using isobaric tags for relative and absolute quantitation (iTRAQ)-based quantitative proteomics and two-dimensional differential gel electrophoresis (2D-DIGE), a comprehensive characterization of the proteomes of *A. minutum* and *A. tamutum* was performed to identify the cellular and molecular underpinnings for the dissimilarity between these two species. A total of 1436 proteins and 420 protein spots were identified using iTRAQ-based proteomics and 2D-DIGE, respectively. Both methods revealed little difference (10–12%) between the proteomes of *A. minutum* and *A. tamutum*, highlighting that these organisms follow similar cellular and biological processes at the exponential stage. Toxin biosynthetic enzymes were present in both organisms. However, the gonyautoxin-producing *A. minutum* showed higher levels of osmotic growth proteins, Zn-dependent alcohol dehydrogenase and type-I polyketide synthase compared to the non-toxic *A. tamutum*. Further, *A. tamutum* had increased S-adenosylmethionine transferase that may potentially have a negative feedback mechanism to toxin biosynthesis. The complementary proteomics approach provided insights into the biochemistry of these two closely related HAB-causing organisms. The identified proteins are potential biomarkers for organismal toxicity and could be explored for environmental monitoring.

## 1. Introduction

The genus *Alexandrium* is one of the major harmful algal bloom (HAB)-causing genera in terms of global diversity, magnitude and consequences of blooms [1,2,3]. The proliferation of this dinoflagellate in the marine environment causes deleterious effects to the economy and human health. Most *Alexandrium* species produce Paralytic Shellfish Toxins (PSTs) such as saxitoxin and analogues which can cause paralysis and eventual death [4].

**Figure 1:**
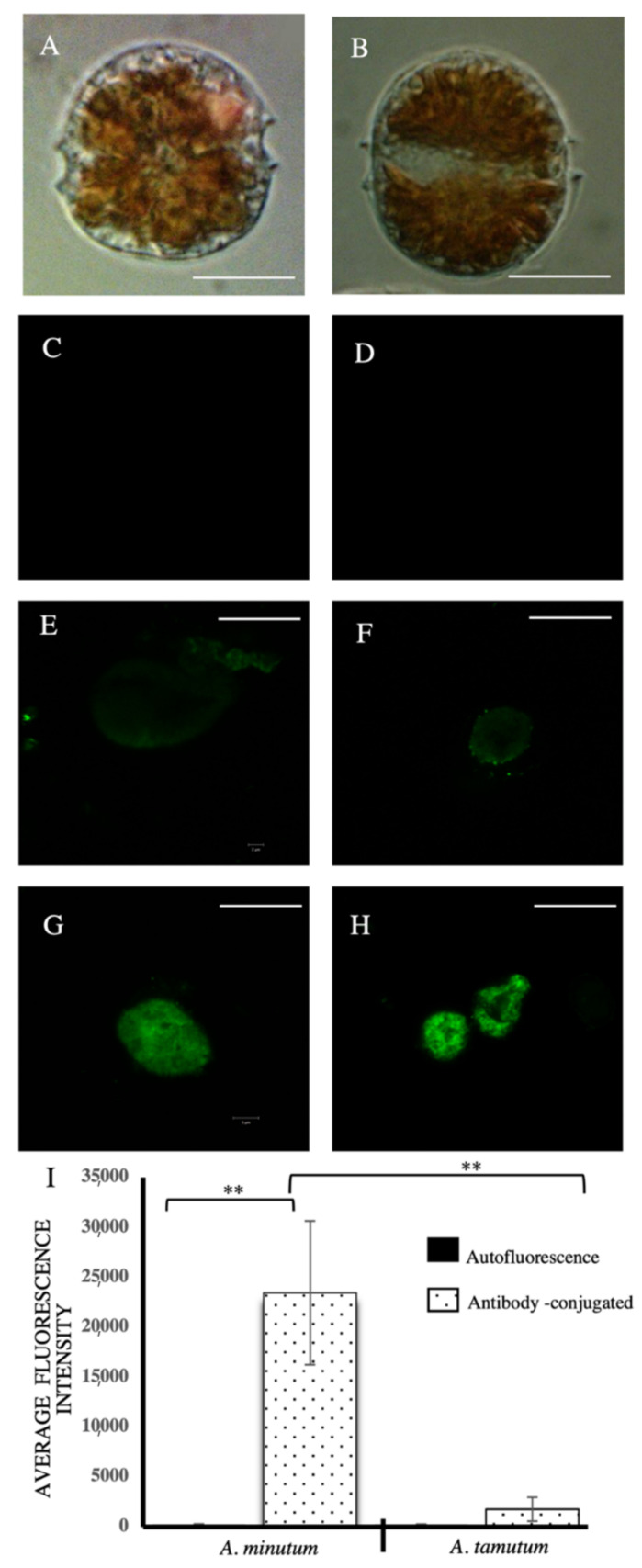
Differentiation of *Alexandrium* species using immunostaining. Antibody raised against *A. minutum* and fluorescein isothiocyanate (FITC)-conjugated goat anti-mouse IgG (H + L) was used as primary and secondary antibody, respectively. Brightfield images (40× magnification) for the closely related *Alexandrium* species, *Alexandrium minutum* (**A**) and *Alexandrium tamutum* (**B**). Scale bar: 20 μm. Negative control for *A. minutum* (**C**) and *A. tamutum* (**D**) after the preparative stages (pigment removal using methanol). Immunostaining of *A*. *tamutum* (**E**,**F**) and *A. minutum* (**G**,**H**). Scale bar: 10 μm at 630× magnification. (**I**) Quantitation of fluorescence intensity for *A. tamutum* and *A. minutum* and negative controls. Immunostaining with primary antibody for *A. tamutum* and *A. minutum*. A significant difference in fluorescence was observed (** *p* < 0.01), with *A. minutum* having the highest fluorescence signal when bound to the *A. minutum*-specific primary antibody. Data is presented as mean ± SD (*n* = 10) and analyzed using ANOVA with post Tukey’s HSD Test (** *p* < 0.01).

*Alexandrium minutum* Halim 1960 and *Alexandrium tamutum* Montresor 2004 are closely related species. Morphologically, both *A. minutum* and *A. tamutum* are oval to elliptical in shape, with the latter slightly pentagonal in some cases. In the cyst stage, *A. minutum* is hemispherical in shape whereas *A. tamutum* is elliptical (Figure 1).

*A. tamutum* and *A. minutum* have comparable cell length between 19–34 µm and 15.5–29 µm, respectively [5,6]. The main diagnostic character between *A. minutum* and *A. tamutum* is that the latter possesses a relatively wide and large sixth precingular plate (6”) [7,8]. This is in contrast with *A. minutum* which has a narrower and smaller sixth precingular plate [6]. The extremely similar size of *A. tamutum* and *A. minutum* often leads to difficulty in cell counting and morphological differentiation.

Phylogenetic differentiation in support of classical taxonomic identification showed the divergence of these two *Alexandrium* species. *A. minutum* and *A. tamutum* form differently well-supported clades based on the nuclear small subunit rDNA and the D1/D2 domain of the large subunit of the large nuclear rDNA [9,10]. In terms of toxicity, *A. minutum* isolates from Australia [11,12], Taiwan [13], Vietnam [14] and Portugal [7] produced gonyautoxin 1,4 (GTX1,4), while an isolate from Italy produced gonyautoxin (GTX2,3) and saxitoxin (STX) as primary toxins [15,16]. Non-toxic *A. minutum* isolates from Ireland and Scotland [17,18] and Italy [19] have also been reported. Meanwhile, various isolates of *A. tamutum* have been reported to be non-toxic [9,10].

Genomics, transcriptomics and proteomics have been utilized for differentiating species, elucidating molecular mechanisms and understanding cellular dynamics among HAB-causing organisms [20]. However, the large genome size and permanently condensed chromosomes of dinoflagellates has impeded the use of genomics for molecular analysis [21]. Furthermore, transcriptomics has the limitation of providing little information on protein activity and fitness for functional and ecological analysis since not all transcripts are translated into functional proteins [22]. Owing to these, proteomics presents an alternative approach to reveal great insights on the various cellular processes among dinoflagellates [23,24]. Dinoflagellate proteomics have been employed to study cell growth and regulation [25], response to environmental stresses [26,27], toxin biosynthesis [2,28], species identification and evolution [29], cell wall and cell surface protein identification [30,31] and symbiotic relationship among these organisms [32]. HAB proteomics has led to the discovery of biomarkers of toxicity [33] and development of antibody-based species identification [34] for HAB species identification and monitoring.

In this work, we differentiated *A. minutum* and *A. tamutum* based on their toxin, immunostaining and proteome profiles. Proteomic studies of these two organisms using gel-based (two-dimensional difference gel electrophoresis or 2D-DIGE) and gel-free approaches (isobaric tags for relative and absolute quantitation or iTRAQ) were complementarily used and provided insights on the biochemical machineries of these related organisms.

## 2. Results

### 2.1. Toxin Analysis

PSTs were quantified using high performance liquid chromatography (HPLC) coupled to fluorescence detection of the oxidation products. From the toxin analysis, *A. minutum* contained 14.05–19.50 fmol STX equiv/cell, with GTX1,4 and GTX2,3 as the primary detectable toxins. On the other hand, no toxin peaks were detected for *A. tamutum* (Supplementary Figure S1).

### 2.2. Immunological Analysis

An immunostaining differentiation between *A. minutum* and *A. tamutum* using an antibody raised against *A. minutum* cell lysates was conducted to assess the biochemical difference between these organisms. This monoclonal antibody, developed by Carrera et al. (2010), was raised in BALB/c mice, with the mouse sera extracted at 10–14 days post-immunization [35]. The monoclonal antibody showed significant and selective staining of *A. minutum* as compared to other *Alexandrium* species such as *A. tamarense* and *A. andersonii* [35].

Average fluorescence intensity for *A. tamutum* was significantly lower compared to *A. minutum* (Figure 1), based on One-Way ANOVA analysis and Tukey’s HSD Test (*p* < 0.01) comparison. Autofluorescence was not observed for either species.

### 2.3. Gel-Free Proteomics Approach

Gel-free comparative proteomics analysis was done by iTRAQ labelling of proteins from *A. tamutum* and *A. minutum* followed by tandem mass spectrometry (MS/MS) analysis. Protein identification was done using MASCOT NCBInr (non-redundant) query which had 90,971,994 sequences and 33,504,913,701 residues available at the time of analysis. The search result gave a total of 154,069 queries with a total of 1436 proteins identified. The majority (93%) of the identified proteins have available protein annotation in UniProt. Out of the 1436 proteins identified, 1266 (88%) of the identified proteins based on UniProt were expressed by both *A. minutum* and *A. tamutum*. Common proteins in these two *Alexandrium* species gave 99% UniProt annotation. In terms of gene ontology of the common proteins, 46% are involved in molecular function, 22% are cellular component and 32% are involved in biological processes. The enzyme classes are oxidoreductases (33%), transferases (26%), hydrolases (16%), lyases (10%), isomerases (4%) and ligases (11%).

**Figure 2:**
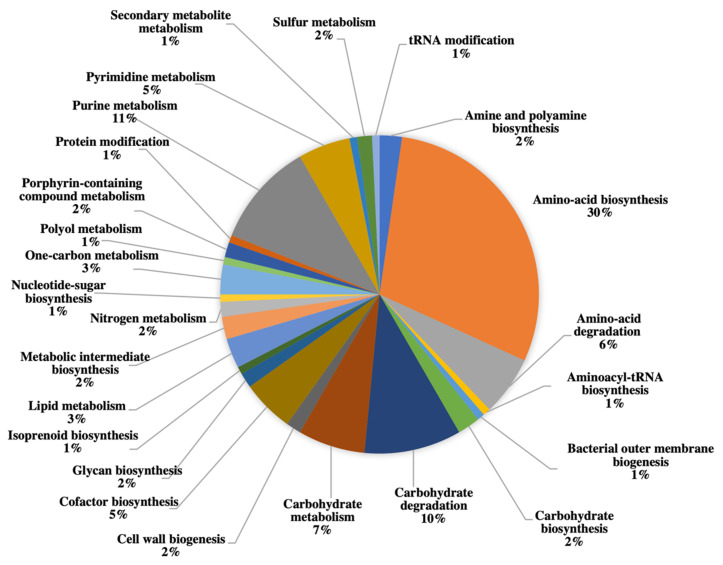
Distribution of identified proteins expressed by both *A. minutum* and *A. tamutum* based on their involvement in biological pathways. The 1,436 identified proteins were annotated using UniPROTKB. Identified proteins are mainly involved in amino acid biosynthesis (30%), purine metabolism (11%), carbohydrate degradation (10%), carbohydrate metabolism (7%) and cofactor biosynthesis (5%).

A breakdown of the proteins identified with biological pathways showed distribution as follows: amino-acid biosynthesis (30%), purine metabolism (11%), carbohydrate degradation (10%), carbohydrate metabolism (7%), amino acid degradation (6%), cofactor biosynthesis (5%), pyrimidine metabolism (5%), lipid metabolism (3%), one-carbon metabolism (3%), amine and polyamine biosynthesis (2%), carbohydrate biosynthesis (2%), cell wall biogenesis (2%), glycan biosynthesis (2%), metabolic intermediate biosynthesis (2%), nitrogen metabolism (2%), porphyrin-containing compound metabolism (2%), sulfur metabolism (2%), aminoacyl-tRNA biosynthesis (1%), bacterial outer membrane biogenesis (1%), isoprenoid biosynthesis (1%), nucleotide-sugar biosynthesis (1%), polyol metabolism (1%), protein modification (1%), secondary metabolite metabolism (1%) and tRNA modification (1%) (Figure 2).

From the 1436 proteins identified in *A. tamumtum* and *A. minutum*, 96 proteins showed varying abundance between the two species based on the iTRAQ labeling approach (Supplementary Table S1).

A significant ratio (*A. tamutum*/*A. minutum* intensity ratio) of 1.3 and 0.77 at 95% confidence interval was used as criteria for high and low protein abundance, respectively.

An interspecies comparison of protein abundance showed 56 proteins were more abundant in *A. minutum* than *A. tamutum*. In contrast, 40 proteins had higher amounts in *A. tamutum*. The more abundant proteins in *A. minutum* were oxidoreductases and hydrolases. On the other hand, abundant proteins in *A. tamutum* were proteins involved in DNA-directed DNA polymerase reactions and acid anhydride catalysis such as 3-hydroxybutyryl-CoA dehydratases. These enzymes are known to be involved in carbohydrate metabolism.

To further improve the protein identification coverage, we conducted a restricted database search using the available in-house mRNA sequence information for the dinoflagellate *Pyrodinium bahamense* (NCBI accession: PRJNA261863, ID: 261863). Protein abundance levels using this reference transcriptome were determined following the significant ratio as mentioned previously.

Utilizing a *P. bahamense* transcriptome sequence data as reference, we identified an additional 19 proteins (Supplementary Table S2) with differential abundance in the two *Alexandrium* species.

The abundant proteins in *A. minutum* include osmotic growth proteins, type 1 polyketide synthase and Zn-dependent alcohol dehydrogenase, serine/arginine splicing factor 6, mitochondrial ubiquinol cytochrome c oxidoreductase core beta subunit-like protein 5, and contig06626_2 and contig00658_4 which both have no annotations and no conserved domains (Table 1). More abundant proteins for *A. tamutum* include ATP synthase subunit, 3-hydroxyacyl-CoA dehydrogenase, photosystem II protein D2, light-harvesting protein, eukaryotic translation initiation factor, methionine synthase, S-adenosyl-methionine transferases, heat shock protein 90, plastid C1 class II fructose bisphosphate aldolase, malate dehydrogenase, and the remaining numbers included predicted, uncharacterized and hypothetical proteins (Table 1).

Other proteins involved in toxin biosynthesis such as argininosuccinate synthase, S-adenosylhomocysteine hydrolase, S-adenosylmethionine synthase, histidine kinase YycG, ribose-5-phosphate, aminomethyltransferase, ferredoxin II, RHS repeat-associated core domain protein, adenylate kinase, transposase, methyltransferase, and ATP sulfurylase/adenylylsulfate kinase (apsK) were identified (Table 2) in both organisms but did not show any significant difference in abundance.

### 2.4. Gel-Based Proteomics Approach

**Figure 3:**
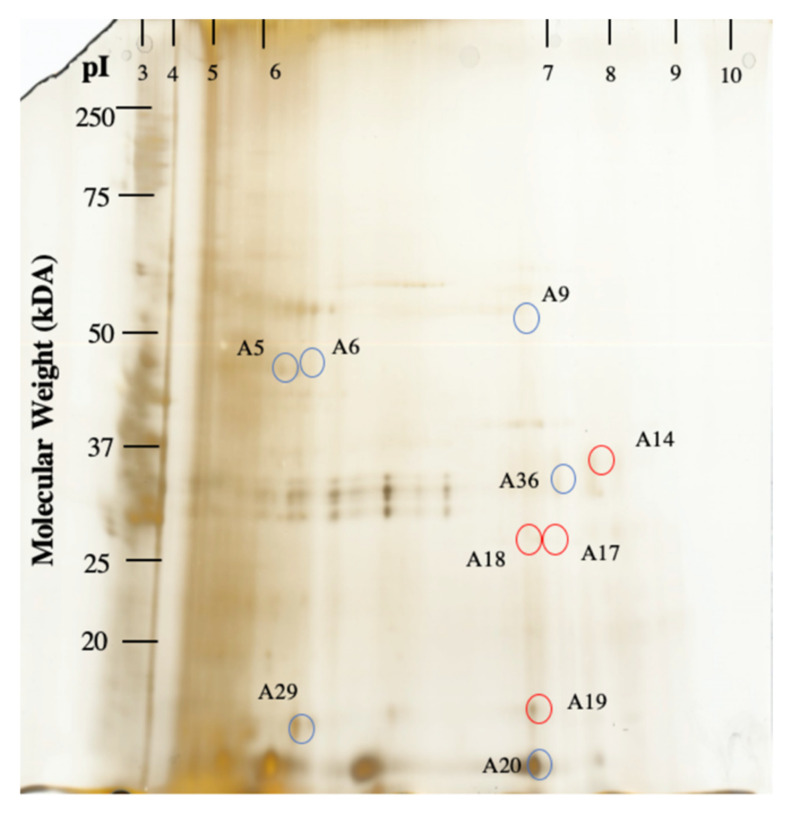
Two-dimensional gel separation of *A. tamutum* and *A. minutum*. *A. minutum* was labeled with Cy3 while *A. tamutum* was labeled with Cy5 (Supplementary Figure S2). Proteins were separated in the first dimension using an immobilized pH gradient isoelectric focusing pI 3–10. Proteins were further separated in the second dimension based on molecular weight by SDS-PAGE. 2D gels were subjected to silver staining. Ten manually excisable protein spots were identified by tandem mass spectrometry (MS/MS). Protein spots with increased amounts in *A. tamutum* and *A. minutum* are labelled with red and blue circles, respectively.

A 2D-DIGE experiment was utilized as a complementary method to determine potential species-specific proteins (Supplementary Figure S2). A total of 420 protein spots were identified for *A. minutum* and *A. tamutum*. Out of these, 41 protein spots (10%) were observed to have >2 intensity ratio, indicative of differing abundance. A total of ten protein spots were prioritized for further analysis based on the signal intensity ratio of >2 and protein spot size (~2 mm diameter size) as criteria for selection (Figure 3).

This was set to ensure successful protein identification after potential protein losses that may arise from extraction and handling since other protein spots were too small for excision. These spots were excised and further analyzed using MS/MS analysis for identification.

In the gel-based approach, high abundant proteins for *A. minutum* include ribulose biphosphate carboxylase (Rubisco), histidine kinases and hypothetical proteins (Table 3). On the other hand, hypothetical proteins, uncharacterized proteins, tRNA modification GTPase and thiamine monophosphate kinase (Table 3) were present in high amounts in *A. tamutum*.

## 3. Discussion

The dinoflagellate *Alexandrium sp*. has been implicated in harmful algal bloom through the production of paralytic shellfish toxins [36,37] and/or fish kills due to bioactive reagents such as reactive oxygen species and polyunsaturated fatty acids [38]. In this study, two *Alexandrium* species from Manila Bay, Bataan, Philippines were characterized. *A. minutum* produced GTX1,4, and GTX2,3 as primary toxins, with similar toxin profiles to *A. minutum* from southern Taiwan [39]. *A. tamutum* did not give any detectable toxins, in agreement with the observed toxin profiles for other *A. tamutum* isolates from the Mediterranean Sea, Italy and Malaysia [9,10]. Reported toxic *Alexandrium* species in the Philippines now include *A. minutum* aside from earlier reported *Alexandrium* cf. *pacificum* and *Alexandrium affine* [2].

Immunostaining significantly differentiated the cells of the two species collected at the exponential phase. The monoclonal antibody used in this experiment specifically binds to an antigen in *A. minutum*. The exact antigen is, however, unidentified since the antibody was raised using whole cell lysate of *A. minutum* [35].

An exhaustive characterization of these two closely related *Alexandrium* species was done using gel-based and gel-free proteomics approaches. A total of 420 protein spots were identified from the gel-based approach, almost three-fold less than the number of proteins identified using the gel-free approach (1436). The low number from 2D-DIGE may be attributed to overlapping and sometimes too small protein spots. The gel-free approach presented a challenge with the de novo peptide sequencing of proteins with very low protein identification scores, often due to the quality of the MS/MS data.

Despite the significant difference in detected proteins in 2D-DIGE and iTRAQ, both approaches showed little variance between the *A. minutum* and *A. tamutum* proteomes. Using the iTRAQ technique, 12% of the identified proteins showed different abundance in both organisms, while 10% of proteins spots were observed to vary using 2D-DIGE.

Our iTRAQ-based proteomic approach yielded comparable results with the gel-free proteomic profiling of *Alexandrium catenella*. Comparison of the protein profiles between the toxic *A. catenella* (ACHK-T) and the non-toxic mutant (ACHK-NT) strain showed 185 differentially expressed proteins from the 3,488 proteins identified (5.3%), with 1.2 and 0.83 significant ratio values as criteria [40]. Moreover, in comparison to a gel-based study conducted by Chan, et al. (2005) [23] for toxic and nontoxic cultured *A. minutum* from Taiwan, proteins were found to vary significantly at pIs ranging from 4.8 to 5.3 with molecular masses between 17.5 to 21.5 kDA using a pI 4–7 strip gradient. We observed differentially expressed proteins at pI of 4–7 using a pI 3–10 gradient and molecular mass of 17–56 kDa. In both studies, only few differences were detected between the toxic and the nontoxic species, in agreement with our observation.

Having the majority of proteins expressed with the same abundance in both organisms suggests that *A. minutum* and *A. tamutum* are most likely to follow similar cellular and biological processes at the exponential stage. This result provides molecular and cellular evidence to the initial study of Figueroa, et al. (2007) [41] which showed that the two species have major common life-cycle patterns and tend to have the same response to modifications in the external nutritional levels. We mapped the proteins with similar abundance levels in the two organisms to biological pathways such as biosynthesis of secondary metabolites, metabolism through carbon fixation, nitrogen metabolism, carbohydrate and lipid metabolism. These are the primary biological pathways involved in the response of the organism to differing nutritional levels in the environment.

Furthermore, proteins identified in both organisms are involved in amino acid biosynthesis, purine metabolism, carbohydrate metabolism, amino acid degradation, co-factor biosynthesis, pyrimidine metabolism, lipid metabolism and one-carbon metabolism such as folate metabolism. As cells are actively dividing at the exponential phase, much of the energy is allocated to the biosynthesis of amino acids. Amino acids are essential building blocks for a variety of biological pathways through cofactor activation as cellular component of new cells, as nutrients for the organism and as energy source [42]. In addition, pyrimidine and purine metabolism are involved in biosynthesis of DNA across all life forms [43]. Processes involving carbohydrates are essential for cellular compartment of the organism and for energy source. Both organisms have thecal plates or “armor” that consist primarily of cellulose [44]. Lipid metabolism among dinoflagellates is essential in adapting to the changing environmental conditions through the alteration of the lipid composition [45]. Folate-mediated one-carbon metabolism is typical among actively dividing eukaryotes as this process supplies one-carbon units to biological pathways such as nucleic acid biosynthesis, mitochondrial and chloroplast biosynthesis, methyl group biogenesis, vitamin metabolism and amino acid metabolism [46].

Proteins with differing abundance in *A. minutum* and *A. tamutum* are involved in vitamin and toxin biosynthesis and osmotic response. Furthermore, our results agree with other proteomic studies which identified differing abundance of proteins related to toxin production such as rubisco [47], alcohol dehydrogenase and S-adenosylmethionine transferase [48].

The gel-based approach showed proteins involved in photosynthesis such as rubisco and histidine kinases being more abundant in *A. minutum*. Rubisco, which among dinoflagellates is encoded by the nuclear DNA instead of the chloroplast DNA, is a protein mainly involved in photosynthesis [49]. In a study of Jiang, et al. (2015) [50], they observed that proteins involved in photosynthesis were upregulated during toxin production. However, the cause-effect relationship has yet to be established. Histidine kinases are transferases that play a major role in signal transduction across cellular membranes. Histidine kinases were recently discovered to be important for alphaproteobacteria and algae interaction via quorum sensing [51].

Abundant proteins for *A. tamutum* include thiamine monophosphate kinase, a key enzyme for vitamin B1 (thiamine) synthesis and converts thiamine monophosphate into thiamine pyrophosphate (coenzyme B1). Vitamin B1 is often derived from de novo synthesis or from the unphosphorylated thiamine (vitamin B1). Most harmful algal bloom species are known to be vitamin B1 and B2 auxotrophs or organisms that are dependent from available nutrients in the environment [52]. The prevalence of photosynthetic microalgae dependent on vitamin auxotrophy are as follows: 22% require vitamin B1 (thiamine), approximately 5% require biotin (vitamin B7), and more than 50% require vitamin B12 (cobalamin) [53,54]. Tang, et al. (2010) [52] showed that *A. minutum* CCMP113 from Vigo, Spain does not require thiamine from its environment for its proliferation. In this study, we report the presence of thiamine monophosphate kinase in *A. tamutum*. Interestingly, Cruz-Lopez and Maske (2016) [55] showed that in some marine dinoflagellates, vitamin B1 and vitamin B12 are provided by the associated bacteria. This indicates the potential role of endosymbionts in *A. tamutum* vitamin B1 production. The role of differential quantities of vitamin B1 and vitamin B12 has been implicated in toxin production and regulation. Since the cultures used in this study are xenic, utilization of axenic cultures can be explored to assess whether these dinoflagellates are prototrophic or auxotrophic in relation to the mentioned vitamins.

In terms of toxin-biosynthesis, the present study was able to identify proteins involved in toxin production in *A. tamutum* and *A. minutum*. Further, this proteomic result corroborates with the recent study of Vigniani, et al. (2020) in which transcripts related to saxitoxin-biosynthesis were detected in the non-toxic clone of *A. tamutum* isolated in Italy [56]. However, most of these detected proteins did not significantly differ in abundance (Table 2). This parallels the results of Zhang, et al. (2015) [40] for the toxic and nontoxic strains of *A. catenella* where only a small proportion of proteins involved in toxin synthesis were observed to vary.

Among the abundant proteins in *A. minutum*, type I polyketide synthase and alcohol dehydrogenases are directly involved in PST biosynthesis. Alcohol dehydrogenases are involved in the reduction of the terminal aldehyde in the eighth step of the predicted saxitoxin biosynthesis [57], while type I-polyketide synthase is involved in the initiation of toxin biosynthesis. In addition, osmotic growth protein, which catalyzes the reduction of fumarate to succinate and essential for anaerobic growth, was also observed to be more abundant in *A. minutum*. The osmotic growth protein enables dinoflagellates to grow despite the osmotic pressure from the environment [58,59]. Errera and Campbell (2011) [60] showed that osmotic stress triggered brevetoxin production in *Karenia brevis*. It was observed that the rapidly changing salinity which simulate a shift from oceanic conditions to a decreased salinity and mimics coastal conditions triggered a 14-fold increase in brevetoxin cell quota while the growth rate remain unchanged. However, this claim that osmotic pressure leads to toxin production has been challenged by other researchers [61]. The observed upregulation of histidine kinase and type-I polyketide synthase in this study parallels the results of Wang et al. (2012), where these proteins showed higher abundance in the toxic *A. catenella* compared to the non-toxic clone [28]. The decreased levels of these proteins in non-toxic *Alexandrium* species may limit the available biosynthetic precursors of PSTs and affect the rate of toxin biosynthesis. Detecting histidine kinase and type I-polyketide synthase as differentially expressed proteins in multiple toxic and non-toxic *Alexandrium* species is suggestive of the potential utility of these proteins to serve as biomarkers for toxin production. However, in the follow up study of Zhang et al. (2015), there was no significant difference in the expression of toxin-related enzymes and instead, differential toxicity was related to carbon and energy utilization [40].

**Figure 4:**
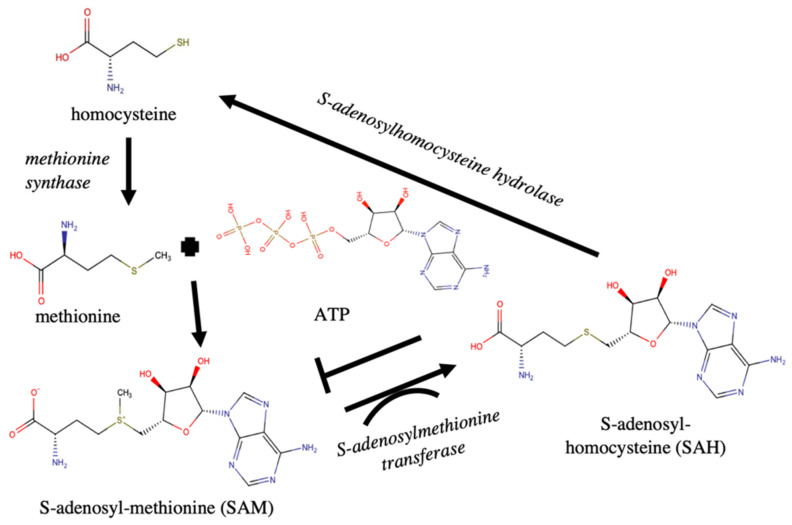
Feedback mechanism for the synthesis of S-adenosyl-methionine (SAM) and S-adenosylhomocysteine (SAH). The observed high abundance of S-adenosylmethionine transferase may favor the reaction for SAH. Excess SAH may lead to inhibition of methylation which is important for toxin production. This may provide the basis for the low toxicity in *A. tamutum* despite the presence of several proteins involved in toxin production.

Noticeably, methionine synthase and S-adenosylmethionine (SAM) transferase, proteins involved in the methionine cycle [62], have increased levels in *A. tamutum*. Methionine synthase is responsible for the production of methionine from homocysteine. ATP when reacted with methionine in the presence of S-adenosylmethionine synthase forms SAM. SAM when methylated via S-adenosylmethionine (SAM) transferases becomes S-adenosylhomocysteine (SAH). SAH is reduced to homocysteine and other forms through the action of S-adenosylhomocysteine hydrolases. Despite the high abundance of SAM transferase in *A. tamutum*, there was no significant difference in the abundance of S-adenosylhomocysteine hydrolases, suggesting potential increase in SAH levels. Excessive SAH acts as an inhibitor of methylation reactions because it has a high affinity for most methyltransferases. This feedback mechanism has so far been reported in humans and plants but not in dinoflagellates [63,64]. This initial finding may suggest that the mechanism for SAH production might be a preferred reaction. It is possible that a negative feedback inhibition as a result of higher abundance of the SAM transferases may lead to inhibition of methylation (Figure 4).

Methylation is a key reaction in the initial steps of saxitoxin production. Potential feedback mechanism to toxin biosynthesis has also been proposed for the non-toxic *A. catenella* strain [28], albeit with different set of proteins. In addition, the importance of SAM for toxin biosynthesis has also been highlighted in previous studies [40,48]. The toxic *A. catenella* strain showed increased levels of enzymes related to SAM biosynthesis [40,48] compared to the non-toxic strain. Our findings further support the potential contribution of feedback mechanisms to toxin production and warrant further investigation. Additional experiments using these proteins as target are suggested to probe the effect of these enzymes on toxin production.

## 4. Conclusions

An exhaustive comparative quantitative proteomics enabled the characterization and differentiation of two closely related *Alexandrium* species. Biochemical machineries such as toxin biosynthesis-related proteins were expressed by both organisms. The difference in abundance levels may possibly lead to changes in cellular dynamics and consequently, affects PST biosynthesis. A possible feedback mechanism is proposed. The favored reaction towards S-adenosylhomocysteine may lead to inhibition of methylation which is an important step in toxin production. Decreased levels of toxin biosynthetic enzymes and other proteins that create feedback mechanisms affecting the availability of PST biosynthetic enzymes and precursors may affect the toxin biosynthesis capability of *Alexandrium* species. Overall, this study has provided insights on the commonalities and divergence between two *Alexandrium* species and provided insights into the critical cellular machinery of these organisms.

## 5. Materials and Methods 

### 5.1. Cultivation and Cell Collection 

Subcultures of *A. minutum* Halim (AminBAT11713) and *A. tamutum* Montresor (Alex2LBol041313) were obtained from the Red Tide Laboratory, Marine Science Institute, University of the Philippines. The initial cultures of the two species were collected from Manila Bay, Bataan, Philippines on 17 January 2013 (*A. minutum*) and 13 April 2013 (*A. tamutum*). Monoclonal cultures were then subcultured in four 4 L flasks with F/2 culture medium under the conditions described previously by Azanza-Corrales and Hall, 1993 [65] and Subong, et al. 2017 [2], with the following modifications: temperature of 24 °C (±2), light intensity of 200 ± 50 µEm^−2^s^−1^ following a 12 h:12 h light: dark cycle. Cell counts were taken every 4–5 days to monitor growth. Starting cell density was normalized to ~200 cells/mL. Two biological replicates at the exponential phase were performed for each species.

### 5.2. Toxin Analysis

Two 50 mL-aliquots of *A. mimutum* and *A. tamutum* cultures were collected at the exponential phase using vacuum filtration with a 0.2 µM nylon filter. These were subjected to cell counting and toxin analysis. *A. tamutum* and *A. minutum* cells were extracted with 0.01 N HCl to obtain the paralytic shellfish toxins (PSTs) extract, as described by Oshima et al. (1995) [66]. PST concentration was determined using the pre-oxidation HPLC-fluorescence detection (HPLC-FD) method described by Lawrence and Ménard (1991) [67,68].

Periodate analysis was done for saxitoxin (STX), neo-saxitoxin (neoSTX) and gonyautoxin 1, 4 (GTX1,4) detection. In brief, a 50 µL aliquot of the PST extract was reacted with 0.03 M H_5_IO_6_, 0.3 M Na_3_PO_4_ and 0.3 M NH_4_HCO_2_ (1:1:1) for 3 min prior to the addition of concentrated CH_3_COOH. The resulting product was analyzed by HPLC-FD. STX, dcSTX and GTX2,3 content was analyzed after peroxide oxidation of the PST extract. The same volume of PST extract was reacted with 1.0 M NaOH and 10% H_2_O_2_ (10:1), and concentrated glacial CH_3_COOH was added after 2 min. The resulting peroxide oxidation product was analyzed by HPLC-FD. HPLC-FD was done using analytical C18 (Inertsil ODS-3V, 250 × 4.6 mm, 5 μm) with 1.0 M NH_4_HCO_2_ (pH 6.0) (Solvent A) and 5 % acetonitrile (Solvent B) as mobile phase. The oxidation products were eluted using the following mobile phase gradient: 1% Solvent B for 2 min; 5% Solvent B for 6 min; 6% Solvent B for 5 min; 10% Solvent B for 10 min at a flow rate of 1.2 mL/min. Oxidation products were monitored at 330 nm (excitation) and 400 nm (emission). The oxidation products of PSTs gave the following retention times (± 0.5 min): neoSTX- 7.50 min, GTX1,4- 8.50 min, STX- 9.75 min, dcSTX- 10.25 min, GTX2,3-10.50min. Toxin standards were sourced from the Certified Reference Materials Program, National Research Council of Canada, Institute for Marine Biosciences, Halifax, Nova Scotia, Canada.

Saxitoxin equivalent/cell was calculated following Parkhill and Cembella (1999) and Borkman, et al. (2014) [69,70]. Toxicity conversion factors were based on the method of Oshima (1995) [66]. Paralytic Shellfish Toxin (PST) concentrations were quantified by integrating the peak areas and calculated using the following equations:

(1) $$Rf=\frac{\mathrm{average}\;\mathrm{peak}\;\mathrm{of}\;\mathrm{standard}}{\mathrm{standard}\;\mathrm{concentration}\;\times \;V}$$

(2) $$PST\;conc.=\frac{\mathrm{PST}\;\mathrm{peak}\;\mathrm{area}/Rf}{V\;\times \;\mathrm{df}}$$

(3) V ≡ Volume; Rf ≡ retention factor; df ≡ dilution factor

### 5.3. Immunostaining Using Confocal Laser Scanning Microscopy

Immunofluorescence of cultured whole algal cells was performed using the procedure described by Lin and Carpenter (1996) and Carrera, et al. (2010) [35,71] with some modifications. Cells at exponential phase were harvested, collected by centrifugation (500*g* for 5 min, 37 °C) in a refrigerated centrifuge and fixed overnight using 4% paraformaldehyde in 0.1 M PBS. Samples were then rinsed with 0.1 M PBS (3×) and were treated with methanol at −20 °C for 24–72 h to remove the pigments. A final concentration of 5 ×10^4^ cells in PBS/1% BSA for each aliquot was subjected for antibody labeling. Labeling was performed using 50 µL of hybridoma supernatant containing monoclonal antibody (with concentration of ~5–25 mg/mL) for 1 h at room temperature with occasional shaking. The cells were washed with PBS/1% BSA and subsequently incubated with 50 µL of secondary antibody, fluorescein isothiocyanate (FITC)-conjugated goat anti- mouse IgG (H + L) (1:200 in PBS/1% BSA). The samples were incubated in the dark for 1–2 h at room temperature and subsequently washed with PBS/1% BSA. The samples were resuspended in 200 µL of PBS/1% BSA. Finally, the cells were imaged using a 630× magnification of a confocal laser-scanning microscope (Zeiss LSM 710, Carl Zeiss AG, Oberkochen, Germany). Two technical replicates were done per biological replicate. A negative control was also performed, with no antibody labeling to assess potential pigment fluorescence. For the single cell analysis of immunofluorescence, 5–10 cells for each sample per biological replicate were imaged for each experiment. Images were then processed for green intensity quantitation using ImageJ (Bankhead, 2014) [72] bundled with Java 1.80.0 software (US National Institutes of Health, Bethesda, MD, USA). The antibody was a generous gift from Prof. Africa Gonzales-Fernandez of Area de Inmunologia, Universidad de Vigo, Spain.

### 5.4. Gel-Free Proteomics

#### 5.4.1. Protein Extraction for iTRAQ-Method

A total of 3 × 10^6^ cells for each sample was used for protein extraction. Samples were lysed (burst of 5 s) using a sonicator probe for 5 min and cell lysis was confirmed by visual inspection using a microscope. The lysis buffer contained 6 M urea, 3 M thiourea, 20 mM TEAB, 5 mM TECP, 0.1% SDS, and 0.1% protease inhibitor mix. Cell lysates were centrifuged at 5000*g*, 4 °C for 5 min. The supernatants were recovered and precipitated using 20% TCA/acetone precipitation to yield the protein pellets. The protein pellets were washed with acetone (3×) to remove residual TCA. Protein pellets were resuspended in 9 M urea and 0.5% SDS and protein concentrations were measured using Bradford assay. In total, two biological replicates were performed.

#### 5.4.2. Protein Labelling and Strong Cation Exchange Chromatography

For each biological replicate, one technical replicate was provided for iTRAQ analysis. Protein samples (50 µg) were reduced using DTT, alkylated with iodoacetamide and digested with trypsin. Tryptic digests were desalted using C18 zip-tip prior to iTRAQ 4-plex (AB Sciex, Foster City, CA, USA) labeling. *A. minutum* was labeled with 114 and 117 isobaric tags while *A. tamutum* with 115 and 116 isobaric tags. Labeled samples were combined in a 1:1:1:1 ratio (with BSA as internal control) and prefractionated offline using strong cation exchange column, polysulfoethyl A (200 × 2.1 mm; 5 µm) (PolyLC INC., Columbia, MD, USA) column, on an Agilent 1100- HPLC system (Agilent Technologies, Palo Alto, CA, USA). Solvent A was: 25% CH_3_CN; 0.1% HCOOH while Solvent B was 25% CH_3_CN; 0.1% HCOOH; 500 mM KCl. A linear gradient from 0–100% Solvent B in 45 min was performed with flow rate set at 200 µL/min. Peptides were detected using a UV detector set at λ = 214, 260 and 280 nm. A total of 21 fractions were collected using a fraction collector. Fractions were further analyzed using High-Performance Liquid Chromatography-Mass Spectrometry (HPLC-MS).

#### 5.4.3. Mass Spectrometry Analysis, Protein Identification and Annotation

Collected fractions were dried and resuspended in loading buffer for reversed phase chromatography coupled to an LTQ-Orbitrap XL mass spectrometer. Mass spectrometry analysis was performed at Proteome Factory AG (Berlin, Germany). The LC component comprised of an Agilent ZORBAX SB-C18 (150 × 0.075 mm; 3.5 µm) column attached to an Agilent 1100 HPLC system (Agilent Technologies, Palo Alto, CA, USA). Fractions 1–7 and 20–21 were analyzed using short gradients (60 min each) while the remaining fractions were analyzed using a long gradient (240 min). Solvent A is 5% CH_3_CN + 0.1% HCOOH while the Solvent B is 100% CH_3_CN + 0.1% HCOOH. The gradient for the 60 min run consisted of 5% Solvent B for 5 min, 5–32% Solvent B in 50 min, 32%–90% Solvent B in 1.5 min, 90% Solvent B for 4 min. The gradient for the 240 min run includes 5% Solvent B for 5 min, 5%–20% Solvent B in 190 min, 20%–32% Solvent B in 30 min, 32%–90% Solvent B in 3 min, 90% Solvent B for 6 min. The MS method was performed following a data-dependent acquisition with MS-overview scan (MSI) between 350–16,000 m/z with 30,000 resolution. This was followed by MS/MS fragmentation of top five ions with collision-induced dissociation (CID) fragmentation at collision energy of 35 eV and fragmentation of the same precursor ions with higher-energy collisional dissociation (HCD) at normalized collision energy of 45 eV. The signal intensity threshold was set to 20,000, isolation width of 3 Da m/z and activation of 30 ms. Ions with unassigned charged states and singly charged were rejected for data dependent acquisition. Dynamic exclusion was activated as follows: 2 repeats in 30 s (top 5 most intense ions fragmented twice in CID and HCD) with exclusion list size 500, exclusion duration 240 s and exclusion mass width of 40 ppm. Tune file information include AGC target FTMS full scan of 2 × 10^5^, maximum injection time of 500 ms, AGC target MS^n^ ion trap: 1 × 10^4^; maximum injection time: 500 ms, AGC target MS^n^ FTMS: 1 × 10^5^, maximum injection time of 500 ms. All scans were performed using only one micro scan.

Results were queried against MASCOT, v.2.5 using NCBInr (November 2016) to map the proteomes of *A. minutum* and *A. tamutum*. Subsequent focused database queries against in-house transcriptome sequencing data of *Pyrodinium bahamense* at the Marine Science Institute, University of the Philippines using MaxQuant v1.4 [73,74] was also performed to determine proteins with differing abundance in the two *Alexandrium* species. This was performed to increase the number of identifiable proteins. In-house genome sequence data is publicly available at NCBI accession: PRJNA261863, ID: 261863.

Protein hits which contained <1% false discovery hits at confidence interval (CI) of 95% are reported. Protein score threshold of 20 was utilized for MaxQuant searches [75] while a protein score of 10 was utilized for MASCOT [76].

A cut-off significant ratio (*A. tamutum*/*A. minutum*) of ≥1.3 (CI of 95%) and ≤0.77 was used to describe high or low abundance proteins in *A. tamutum*. Data normalization was performed using the average of the median intensities for the different labels.

Proteins were annotated using UniProt, Gene Ontology (GO) and KEGG protein databases [77,78,79].

### 5.5. Gel-Based Proteomics

#### 5.5.1. Sample Extraction and Preparation for 2D-DIGE

A total of 3 × 10^6^ cells were lysed using a sonicator probe with a buffer containing: 6 M urea, 2 M thiourea, 4% CHAPS, 5 mM MgOAc, 10 mM Tris pH 8.5 with protease inhibitor cocktail (1% v/v). Homogenization was performed on ice and was performed in bursts of 5 s. Samples were transferred to 15 mL tubes and proteins were precipitated using cold 10% TCA in acetone for 1 h. Protein pellets were washed with 80% 0.1 M NH_4_OAc dissolved in 80% CH_3_OH and 20% water (2×) and a final wash of 80% acetone. The air-dried pellets were dissolved in DIGE compatible buffer consisting of 6 M urea, 2 M thiourea, 4% CHAPS, 5 mM MgOAc, 10 mM Tris pH 8.5. Sample was cleaned using a 2D clean up kit according to manufacturer’s protocol (GE Healthcare). The pellets were dissolved in previously mentioned DIGE compatible buffer. Protein content was done with Quick Start Bradford Assay from Bio-Rad according to the manufacturer’s protocol.

#### 5.5.2. 2D-DIGE Labeling and 2D Runs

For 2D-DIGE experiment, 50 µg of *A. minutum* protein was labeled with 250 pmolar Cy3 (Chromis DGE Minimal Labelling Kit from Cyanagen, Bologna, Italy) while 50 µg of *A. tamutum* was labeled with 250 pmolar Cy5 (Chromis DGE Minimal Labelling Kit from Cyanagen, Bologna, Italy) according to manufacturer’s protocol. Reciprocal labeling was also performed. Two technical replicates for each of the two biological replicates were done.

Isoelectric focusing was performed using a 13 cm pH 3–10 NL immobiline strips from GE-Healthcare using a Protean i12 IEF Cell, Bio-Rad for a total 49,520 volt-hours. After first dimension separation, each strip was equilibrated with about 12 mL equilibration buffer (50 mM Tris pH 8.8, 6 M urea, 30% glycerol, 2% SDS, 1% DTT, and trace amount of bromophenol blue) for 10 min and subsequently, equilibrated in fresh equilibration buffer (2.5% iodoacetamide, 50 mM Tris pH 8.8, 6 M urea, 30% glycerol, 2% SDS, 2.5% iodoacetamide, and trace amount of bromophenol blue) for 10 min.

For second dimension separation, 12% SDS-PAGE gels (16 × 15 cm) were used. Gels were run using the following parameters: 20 mA/gel for 20 min followed by 40 mA/gel for a remaining time until the dye front leaves the gel.

Gels were scanned using a Typhoon 9400 fluorescent scanner using wavelengths adequate to Cy3 and Cy5 dye as per manufacturer specification. The resolution of the scan was 100 μm pixel size. Images were then loaded into DIA module (Differential in gel Analysis) of the DeCyder 5.2 software (GE Healthcare Life Sciences, Marlborough, MA, USA). Normalization was performed by the generation of a “master gel” from all the runs including reciprocal labeling runs. Distinct spots were identified following a Gaussian fitting (CI at 95%). Silver staining procedure that is compatible for MS analysis was used as a post-staining procedure. The staining reaction was developed in 30 s. Protein spots were then manually excised for further analysis.

#### 5.5.3. Mass Spectrometry Analysis, Protein Identification and Annotation

Protein samples were trypsin digested and peptides were extracted. In brief, sufficient amount of trypsin (12.5 mg/mL trypsin, 25 mM ammonium bicarbonate) was added to the gel pieces and these were incubated at 37 °C. The digested peptides were extracted (3×) with CH_3_CN containing 1% TFA. The pooled extracts were dried by rotary evaporation and stored at −20 °C until further analysis. Mass spectrometry analysis was performed by 1st BASE (Selangor, Malaysia). Peptides were analyzed by electrospray ionization mass spectrometry with subsequent MS/MS fragmentation using a Shimadzu Prominence nano HPLC system (Shimadzu, Kyoto, Japan) coupled to a high resolution 5600 TripleTOF mass spectrometer (AB Sciex, Framingham, MA, USA). Tryptic peptides were loaded onto an Agilent Zorbax 300SB-C18 (150 × 4.6 mm, 3.5 µm; Agilent Technologies (Palo Alto, CA, USA) and separated with a linear gradient from 0–100% H_2_0/CH_3_CN/0.1% HCOOH (v/v) at a flow rate of 0.5 mL/min for 45 min. Protein identification was done using MASCOT, v2.5 sequence matching software (Matrix Science) with MSPnr100 database (December 2016). The following parameters were used: peptide tolerance: ±0.2 Da, MS/MS tol: tol: ±0.2 Da, peptide charge: 2+, 3+ and 4+, Mass: monoisotopic, Enzyme: trypsin, missed cleavage: 1, fixed modification: carbamidomethylation, variable modification: oxidation (methionine). A protein score of >60 at a CI of 95% was considered as a good hit and was utilized as one of the criteria for gel-based protein identification [2,24,80].

## Figures and Tables

**Table 1:** Proteins with Varying Abundance in *A. tamutum* and *A. minutum* based on iTRAQ Approach and *Pyrodinium bahamense* (Pbc) Transcriptome as Reference.

Protein	Number of Significant Sequences	Sequence Coverage (%)	Representative Ratio (A.T/A.M.) *^a^*	Score	E-Value	Function
Osmotic Growth Proteins [Pbc transcript]	6	27	0.196	392	3.2E ^−39^	succinate dehydrogenase activity
Type 1 Polyketide Synthase ** Sequence: R.SPASTGQSLGR.R [Pbc transcript]	1	4	0.517	40 *	0.00050	polyketide synthesis
Serine/Arginine Splicing Factor 6 ** Sequence: R.GGLAQGSPRP [Pbc transcript]	1	3	0.746	29 *	0.0063	mRNA splicing
Mitochondrial Ubiquinol Cytochrome C Oxidoreductase Core Beta Subunit-Like Protein 5 ** Sequence: R.GIPPAEMLLR.I [Pbc transcript]	1	4	0.772	45 *	0.00016	redox reaction
Contig06626_2 ** Sequence: K.LAGVDAK.D [Pbc transcript]	1	3	0.431	53 *	2.5E ^−05^	No annotation
Contig00658_4 ** Sequence: R.SVVQLLR.R [Pbc transcript]	1	2	0.514	87	10E ^−09^	No annotation
ATP Synthase Subunit [Pbc transcript]	9	22	1.30	1173	2.5E ^−117^	ATP production
3-Hydroxyacyl-CoA Dehydrogenase [Pbc transcript]	3	18	1.36	117	10E ^−12^	redox reaction
Photosystem II protein D2 [Pbc transcript]	5	14	1.38	87	10E ^−09^	photosynthesis
Light-Harvesting Protein [Pbc transcript]	2	9	1.38	215	1.6E ^−21^	photosynthesis
Eukaryotic Translation Initiation Factor [Pbc transcript]	3	8	1.56	154	2.0E ^−15^	translation
Methionine Synthase ** Sequence: K.GLNMSIVNPGGLPR.Y [Pbc transcript]	1	5	1.84	27 *	0.010	methionine production
Heat Shock Protein 90 [Pbc transcript]	6	8	2.10	67	10E ^−07^	protein stabilization against heat stress
Plastid C1 Class II Fructose Bisphosphate Aldolase [Pbc transcript]	3	8	2.30	206	1.3E ^−20^	glycolytic process
Malate Dehydrogenase ** Sequence: R.SVLAGLSGR.K [Pbc transcript]	1	2	2.31	84	2.0E ^−08^	citric acid cycle
Zn-Dependent Alcohol Dehydrogenase ** Sequence: K.LNTGITPLEVAPMADAGITAYR.A [*Marinovum Algicola*]	1	16	0.456	153	2.5E ^−15^	aldehyde production
Type-I Polyketide Synthase Sequence: R.SPASTGQSLGR.R [Pbc transcript]	1	3	0.517	40 *	0.0005	polyketide synthesis
S-Adenosyl-Methionine Transferases [Pbc transcript]	4	9	3.846	314	2.0E ^−31^	methylation

*^a^*A.T. and A.M. refers to *A. tamutum* and *A. minutum*, respectively. * denotes MaxQuant Score; otherwise, MASCOT score is reported; ** unique peptide.

**Table 2:** Toxin Biosynthesis-Relevant Proteins with Consistently Non-varying Abundance in *A. minutum* and *A. tamutum.*

Protein	Number of Significant Sequences	Sequence Coverage (%)	Score	E-Value	Function
Argininosuccinate Synthase [*Ruegeria atlantica*]	2	10	72	3.2E ^−07^	urea cycle
S-Adenosylhomocysteine Hydrolase ** Sequence: R.ATDVMIGGK.R [*Alexandrium*]	1	6	109	6.3E ^−11^	adenosine and homocysteine production
S-Adenosylmethionine Synthase [Mameliella Alba]	2	7	71	4.0E ^−07^	SAM synthesis
Histidine Kinase YycG ** Sequence: K.NPLASLRSAVGSLR.M [*Mameliella Alba*]	1	1	52 *	3.1E ^−05^	signal transduction
Aminomethyltransferase ** Sequence: K.AGLMDVSGLK.K [Ruegeria Atlantica]	1	3	50 *	0.00005	methylenetetra-hydrofolate catabolism
Ferredoxin II ** Sequence: K.FSEQWPVIVTK.K [*Ruegeria Atlantica*]	1	12	35 *	0.0020	electron transfer
RHS Repeat-Associated Core Domain Protein ** Sequence: R.ATWAPGAAGDWQSTGGMLTNAGATGPATLTAATADPAR.G [*Mameliella Alba*]	1	3	34 *	0.0020	bacterial exotoxin
Adenylate Kinase ** Sequence: R.TLAQADALDALLAK.H [*Marinovum Algicola*]	1	8	33 *	0.0030	cellular energy homeostasis
Transposase ** Sequence: R.DWIGAVGAK.T [*Ruegeria Atlantica*]	1	23	25 *	0.020	transposon transporter
Methyltransferase** Sequence: R.ITTGVGK.G [*Marinovum Algicola*]	1	4	23 *	0.030	methylation
ATP Sulfurylase/Adenylylsulfate Kinase (apsK) ** Sequence: K.VYLGGPVTGIQQPVHYDFR.G [*Marinovum Algicola*]	1	3	20 *	0.05	activated sulfate synthesis

* denotes MaxQuant Score; otherwise, MASCOT score is reported; ** unique peptide.

**Table 3:** Proteins with Differential Abundance in *A. tamutum* and *A. minutum* based on 2D-DIGE Method.

Proteins abundant in *A. minutum*							
Protein Spot Code	Protein	Number of Peptides	Sequence Coverage (%)	Average Relative Intensity (A.M./A.T.) *^a^*	Score	E-Value	Function
A5 *	Ribulose Bisphosphate Carboxylase [*Acidithiobacillus Caldus*]	4	13	2.05	93	2.5E ^−11^	carbon fixation
A6 *	Ribulose Bisphosphate Carboxylase [*Acidithiobacillus Caldus*]	4	13	3.84	103	2.5E ^−12^	carbon fixation
A9	Penicillin-Binding Protein [*Parcubacteria Bacterium*]	3	2	3.7	66	1.3E ^−08^	peptidoglycan biosynthesis
A20	Uncharacterized Protein ** Sequence: R.IAAELADGR.R [*Streptosporangium Roseum*]	1	3	8.9	164	2.0E ^−18^	no annotation
A23	Histidine Kinase [*Geobacter Pickeringii*]	3	1	2.81	92	3.2E ^−11^	signal transduction
A36	Uncharacterized Protein [*Streptosporangium Roseum*]	3	3	2.71	68	7.9E ^−09^	no annotation
**Proteins abundant in *A. tamutum***							
**Protein Spot Code**	**Protein**	**Number of Peptides**	**Sequence Coverage (%)**	**Average Relative Intensity (A.T./A.M.) ^a^**	**Score**	**E-Value**	**Function**
A14	Thiamine-Monophosphate Kinase [*Bacteroides Vulgatus*]	3	3	4.11	68	7.92E ^−09^	thiamine biosynthesis
A17	tRNA Modification GTPase MnmE [*Bosea sp.* 117]	2	3	3.27	80	5.0E ^−10^	tRNA methylation
A18	Hypothetical Protein [*Actinoplanes Globisporus*]	2	1	4.64	65	1.6E ^−08^	No annotation
A19	Uncharacterized Protein [*Lautropia Mirabilis* ATCC 51599]	2	2	2.37	68	8.0E ^−09^	No annotation

Legend: Blue Protein Spot Code shows high-abundant proteins for *A.minutum*. Red Protein Spot Code shows high-abundant proteins for *A. tamutum* (downregulated for *A. minutum*). Note: Different isoforms of the same protein, typical for dinoflagellates (Wang, et al., 2011). *^a^*A.T. and A.M. refers to *Alexandrium tamutum* and *Alexandrium minutum*, respectively. ** unique peptide.

## Supplementary Materials

The following are available online at https://www.mdpi.com/2072-6651/13/1/7/s1, Figure S1: Representative toxin chromatogram for *Alexandrium* species, Figure S2: 2D-DIGE images for *A. minutum* and *A. tamutum*, Table S1: Quantification and Identification of Proteins using NCBI, Table S2: Quantification and identification of Proteins using *Pyrodinium bahamense* transcriptome.
